# T-cell activation is an immune correlate of risk in BCG vaccinated infants

**DOI:** 10.1038/ncomms11290

**Published:** 2016-04-12

**Authors:** Helen A. Fletcher, Margaret A. Snowden, Bernard Landry, Wasima Rida, Iman Satti, Stephanie A. Harris, Magali Matsumiya, Rachel Tanner, Matthew K. O'Shea, Veerabadran Dheenadhayalan, Leah Bogardus, Lisa Stockdale, Leanne Marsay, Agnieszka Chomka, Rachel Harrington-Kandt, Zita-Rose Manjaly-Thomas, Vivek Naranbhai, Elena Stylianou, Fatoumatta Darboe, Adam Penn-Nicholson, Elisa Nemes, Mark Hatherill, Gregory Hussey, Hassan Mahomed, Michele Tameris, J Bruce McClain, Thomas G. Evans, Willem A. Hanekom, Thomas J. Scriba, Helen McShane

**Affiliations:** 1 Jenner Institute, Nuffield Department of Medicine, University of Oxford, Oxford OX3 7DQ, UK; 2 Department of Immunology and Infection, London School of Hygiene and Tropical Medicine, London W1CE7HT, UK; 3 Aeras, Rockville, Maryland 20850, USA; 4 Biostatistics Consultant, 1129 N. Illinois Street, Arlington, Virginia 22205, USA; 5 Oxford Vaccine Group, Department of Paediatrics, University of Oxford, Oxford OX3 7LE, UK; 6 Kennedy Institute, Nuffield Department of Orthopaedics, Rheumatology and Musculoskeletal Sciences, University of Oxford, Oxford OX3 7LF, UK; 7 Wellcome Trust Centre for Human Genetics, Nuffield Department of Medicine, University of Oxford, Oxford OX37BN, UK; 8 South African Tuberculosis Vaccine Initiative, Institute of Infectious Disease and Molecular Medicine, Department of Paediatrics and Child Health, University of Cape Town, Cape Town 7935, South Africa

## Abstract

Vaccines to protect against tuberculosis (TB) are urgently needed. We performed a case–control analysis to identify immune correlates of TB disease risk in Bacille Calmette–Guerin (BCG) immunized infants from the MVA85A efficacy trial. Among 53 TB case infants and 205 matched controls, the frequency of activated HLA-DR^+^ CD4^+^ T cells associates with increased TB disease risk (OR=1.828, 95% CI=1.25–2.68, *P*=0.002, FDR=0.04, conditional logistic regression). In an independent study of *Mycobacterium tuberculosis*-infected adolescents, activated HLA-DR^+^ CD4^+^ T cells also associate with increased TB disease risk (OR=1.387, 95% CI=1.068–1.801, *P*=0.014, conditional logistic regression). In infants, BCG-specific T cells secreting IFN-γ associate with reduced risk of TB (OR=0.502, 95% CI=0.29–0.86, *P*=0.013, FDR=0.14). The causes and impact of T-cell activation on disease risk should be considered when designing and testing TB vaccine candidates for these populations.

## Abstract (web summary)

BCG vaccine confers only partial protection against tuberculosis. Here the authors show that the risk of tuberculosis infection and progression to disease in BCG-immunized children positively correlates with the frequency of activated HLA-DR^+^CD4^+^ T cells.

## Graphical abstract

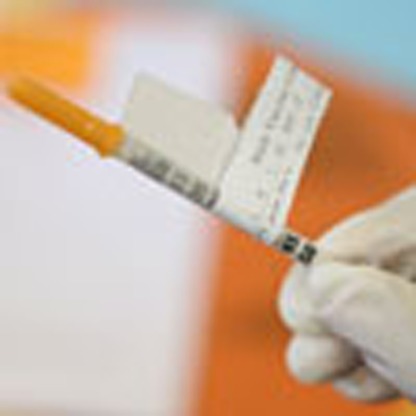

Tuberculosis (TB) causes higher mortality than any other infectious disease globally and there is an urgent need for improved vaccines if we are to control the epidemic. The incidence of TB disease in children under 2 years of age in the Western Cape of South Africa is estimated to be 1.5% per annum^1^. Despite the widespread use of Bacille Calmette–Guérin (BCG), we do not have an immune correlate of TB risk following immunization. Defects in cytokine signalling pathways for interferon (IFN)-γ and interleukin (IL)-12 increase susceptibility to mycobacterial infection in humans^2^,^3^,^4^,^5^,^6^, suggesting a role for these cytokines in protection. HIV infection is also a risk factor, with TB risk increasing as CD4 T-cell counts decline^7^. Animal studies have confirmed the importance of CD4 T cells and IFN-γ in protection from TB disease^8^,^9^ and have guided the development of new candidate TB vaccines, which increase immunity against *M. tuberculosis* through boosting of a Th1 type cellular immune response^10^. However, results from an immune correlates study in BCG-vaccinated infants from the Western Cape of South Africa did not find BCG antigen-specific Th1 cytokine-secreting T cells to be associated with reduced TB disease risk^11^, and the Th1 boosting candidate TB vaccine MVA85A (Modified Vaccinia virus Ankara expressing Ag85A from *M. tuberculosis*) did not improve protection in BCG-vaccinated infants^12^. To aid the development of more effective TB vaccines, there is an urgent need to identify immune correlates of TB disease risk. Although MVA85A did not protect against TB disease beyond that conferred by BCG alone, all infants were vaccinated with BCG at birth and analysis of samples collected from infants subsequently diagnosed as TB cases in the trial provided an opportunity to identify correlates of TB risk^12^. We performed a case–control study on cryopreserved blood samples using immune assays, that measured both BCG-specific and non-specific immune responses with the aim of identifying immune variables, which correlate with disease risk in BCG-vaccinated infants. In BCG-vaccinated infants, activated HLA-DR^+^ CD4^+^ T cells associate with increased risk of TB and BCG-specific T cells secreting IFN-γ associate with reduced risk of TB disease over the next 3 years of life. The causes and impact of T-cell activation should be considered when designing and testing candidate TB vaccines.

## Results

### Primary analysis

**Figure 1:**
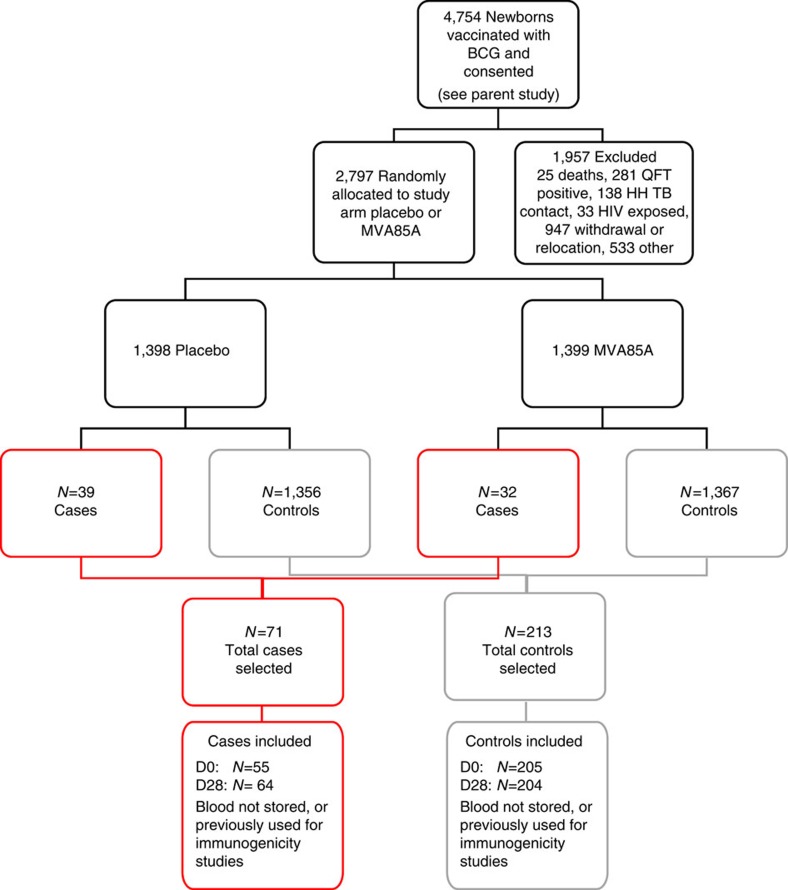
Cohort of infants from the MVA85A efficacy trial included in this immune correlates case–control study. At 4–6 months of age, blood was collected from HIV-negative, HIV unexposed BCG-vaccinated infants with no active or chronic illnesses (including suspected TB), and with no household exposure to an adult who had active TB disease. Infants were then randomized to receive either MVA85A or placebo and followed for 2–3 years. TB cases (*n*=71) were defined by a positive culture for *M. tuberculosis* or the presence of *M. tuberculosis* DNA using a certified molecular diagnostic assay; or a stringent composite end point, which included symptoms, radiological signs and an exposure history. Control infants (*n*=213) had no evidence of TB exposure, no evidence of TB infection on interferon gamma release assay (IGRA) and were matched (3 controls to 1 case) on age, race, time on study and CDC weight percentile.

Blood samples for immunologic assessment were obtained from all infants within 7 days before vaccination with either MVA85A or placebo (D0) and 28 days (D28) following vaccination (Fig. 1). We performed *ex vivo* IFN-γ ELISPOT and mycobacterial growth inhibition assays (MGIAs) to assess antigen-specific T-cell responses, performed ELISA to assess antibody responses and characterized cells using flow-cytometry with cell surface staining for Live/Dead cells followed by staining with CD3, CD4, CD8, CD14, CD16, CD19, CD25, CD127 and HLA-DR.

**Figure 2:**
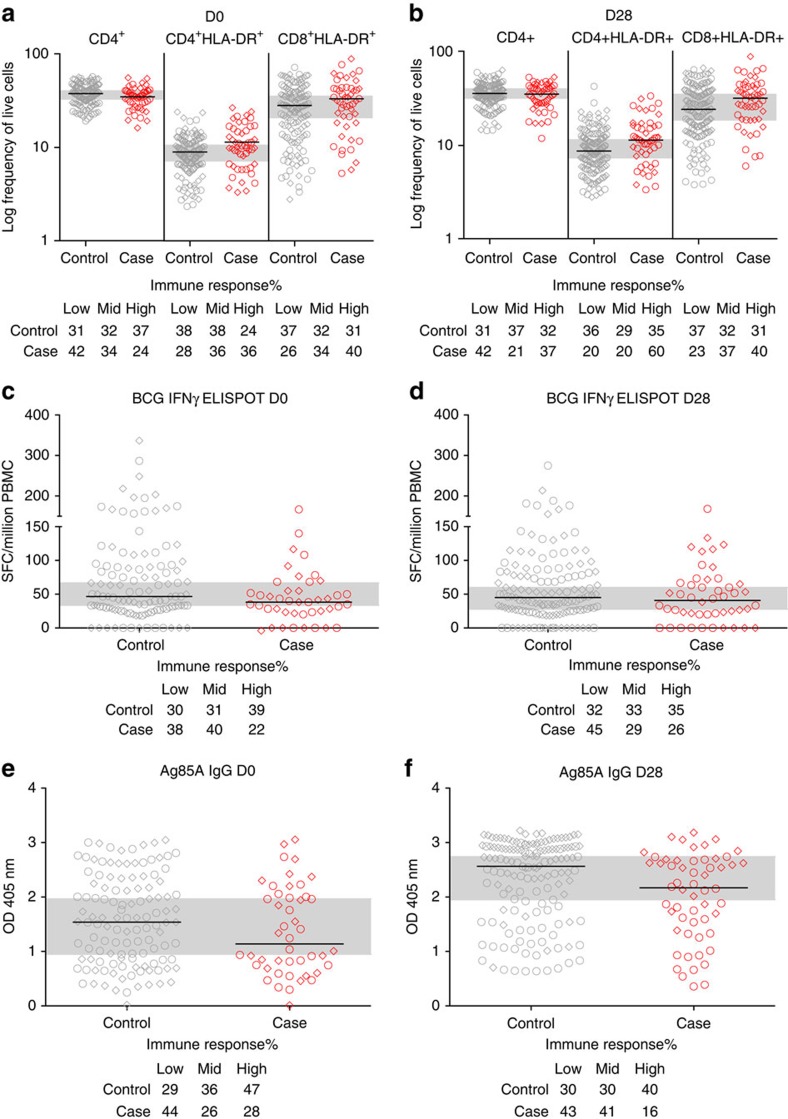
Distribution of significant immune variables in TB case and controls from the infant MVA85A vaccine trial. Immune variables *P*<0.05 at D0 or D28 are shown. Infants are stratified according to TB case (red) or control (grey) status. The 50th percentile of the immune variable is indicated by a horizontal line. The shape of the point indicates whether an infant was in the placebo (round) or MVA85A (diamond) arm of the study. There were no significant differences in immune variables when infants were stratified by study arm. Combined low, medium and high immune responses at D0 (**a**,**c**,**e**) or D28 (**b**,**d**,**f**) were used to divide the all samples (both case and control groups) into thirds; medium third is indicated by the grey horizontal bar. The log frequency of live CD3^+^ cells expressing CD4, CD4 and HLA-DR or CD8 and HLA-DR, respectively. SFC/million PBMC is the number of IFN-γ spot-forming cells per million PBMC (only results from infants with a PHA response greater than 1,000 SFC/million are shown). Optical density was measured by ELISA at wavelength 405 nm.

Conditional logistic regression analysis was used to assess the association between D0 immune response and risk of TB disease. To adjust for multiple comparisons, the false discovery rate (FDR) was calculated for each variable using the method of Benjamini and Hochberg^13^. Of the 22 pre-specified immune response variables evaluated, the frequency of D0 activated HLA-DR^+^ CD4^+^ T cells was associated with increased risk of TB disease (estimated odds ratio (OR) 1.12 per 1 unit increase, *P*=0.002, FDR=0.047; Table 1 and Supplementary Fig. 1). The frequency of D0 CD4^+^ T cells (estimated OR 0.95 per 1 unit increase, *P*=0.027, FDR=0.198) and the magnitude of D0 BCG-specific T cells secreting IFN-γ (estimated OR 0.46 per 1 log10 unit increase, *P*=0.03, FDR=0.198) were associated with reduced risk of TB disease (Fig. 2a,c). For subsequent assessment of linearity of the immune parameters, we classified immune responses into low, medium and high categories based on tertiles of the data (Fig. 2).

**Figure 3:**
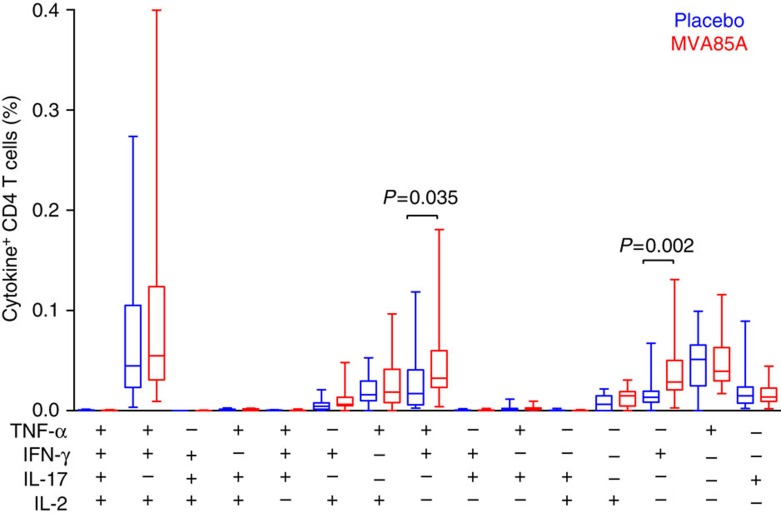
BCG-specific CD4 T-cell response in whole blood. Frequencies of cytokine-expressing BCG-specific CD4-positive T cells expressing IFN-γ, tumour-necrosis factor (TNF)-α, IL-2 or IL-17 measured by whole blood intracellular cytokine staining 28 days after administration of placebo (*n*=19) or MVA85A (*n*=17). Only *P*-values<0.05 shown. Mann–Whitney test, not adjusted for multiple comparisons.

In a whole blood intracellular cytokine staining (ICS) assay, performed on a subset of infants, it was found that the response to BCG was predominantly a CD4^+^ T-cell-mediated polyfunctional response characterized by the production of IFN-γ, tumour-necrosis factor-α and IL-2 (Fig. 3).

Exploratory analysis suggested that D28 post-boost vaccination Ag85A-specific IgG antibodies were also associated with reduced TB disease risk (estimated OR=0.62 per 1 log10 unit increase, *P*=0.019). None of the immune variables were able to predict TB disease risk with an area under the receiver operating characteristic (AUROC) above 0.8. The box-plot of each immune response variable, stratified by case–control status is shown in Fig. 2 and Supplementary Fig. 2. Variables that did not associate with TB disease risk included D0 purified protein derivative (PPD) IFN-γ ELISpot (estimated OR=0.72, *P*=0.453) and D28 Ag85A peptide IFN-γ ELISpot (estimated OR=0.69, *P*=0.183) and the D0 MGIA (estimated OR=0.31, *P*=0.318; Table 1). The magnitude of the IFN-γ ELISpot response to mycobacterial antigens was low. However, cells were functionally able to secrete IFN-γ as ∼25% of infants responded to cytomegalovirus (CMV) stimulation with responses ranging to >1,000 spot-forming cells (SFCs) per million peripheral blood mononuclear cells (PBMCs) (Supplementary Fig. 2). Activated HLA-DR^+^ CD4^+^ and CD8^+^ T cells were highly correlated with each other (*R*=0.53 *P*<0.0001) and both populations were inversely correlated with total CD4^+^ T cells (*R*=−0.427 *P*<0.0001, *R*=−0.391 *P*<0.0001, respectively; Supplementary Table 1). There was a positive correlation between CMV IFN-γ ELISpot and the magnitude of HLA-DR^+^ CD8^+^ T cells (*R*=0.301 *P*<0.0001; Supplementary Table 1).

### Impact of MVA85A vaccination on immune correlates

There was no significant difference by MVA85A or placebo groups at either D0 or D28 when treatment group (MVA85A or placebo) was included as an interaction term in the analysis. Results for HLA-DR^+^ CD4^+^ T cells including treatment group (MVA85A or placebo) as an interaction term in the analysis are shown in Supplementary Table 2.

The day 28 analysis is potentially confounded as Ag85A antibodies are induced by MVA85A immunization and for 8 case infants the matched controls were all from the placebo arm and for 3 placebo infants the matching controls were all from the MVA85A arm. To explore the possibility of any confounding effect of mixed control infants from MVA85A and placebo arms the D28 conditional logistic regression analysis was performed separately on infants only from the MVA85A group and only the placebo group. Results are presented in Supplementary Table 3. There is no statistical significance for any immune variable when infants are analysed separately by study group (likely due to the reduced sample size only ∼25 case infants in each analysis). However, the OR for each immune variable remains in the same direction as reported for the primary analysis.

### Relative contribution to disease risk

To compare the relative effect size of the different immune response variables, we used *z*-score transformation to scale each variable to a standard deviation of 1 and performed conditional logistic regression on *z*-score transformed data (Supplementary Table 4). The OR for Ag85A IgG adjusted from 0.62 to 0.74, for CD4^+^ T cells from 0.95 to 0.65, for BCG ELISpot from 0.46 to 0.502 and for HLA-DR^+^ CD4^+^ from 1.12 to 1.83.

The relative effect of TB disease risk is therefore smallest for Ag85A IgG followed by CD4^+^ T cells, BCG ELISpot and greatest for HLA-DR^+^ CD4^+^ T cells.

### HLA-DR^+^ CD4^+^ T cells in *M. tuberculosis*-infected adolescents

**Figure 4:**
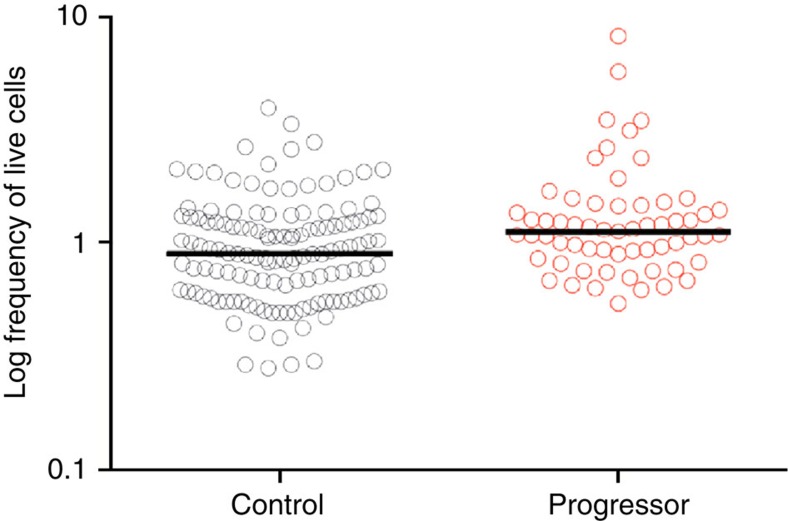
Frequencies of HLA-DR-expressing CD4^+^ T cells in latently *M. tuberculosis*-infected adolescents. Frequencies of HLA-DR-expressing CD4^+^ T cells in *M. tuberculosis*-infected adolescents who progressed to TB disease (red; progressors, *n*=61 samples from 30 progressors) compared with matched controls who did not progress to disease (grey; controls, *n*=132 samples from 59 controls).

We retrieved cryopreserved PBMC from healthy, South African, *M. tuberculosis*-infected adolescents previously enrolled into the Adolescent Cohort Study (ACS)^14^, to determine if frequencies of HLA-DR- expressing CD4^+^ T cells were also associated with risk of progression to TB disease in this independent cohort. HLA-DR-expressing CD4^+^ T cells were measured in 61 samples from 30 adolescents who progressed to microbiologically confirmed incident TB and in 132 samples from 59 matched controls who remained healthy. Overall, frequencies of HLA-DR-expressing CD4^+^ T cells were higher in adolescents who progressed to TB disease compared with matched controls who did not progress to disease OR=1.387, 95% confidence interval (CI)=1.068–1.801, *P*=0.014 (Fig. 4 and Table 2). Using conditional logistic regression, the difference between controls and progressors was significant at all time points before diagnosis of TB disease (Table 2). However, the OR increased at times closer to TB diagnosis (Table 2).

### Risk of TB disease by category of immune response

To assess the linearity assumption of the conditional logistic regression based on a continuous immune response variable, we classified immune responses into low, medium and high categories based on tertiles of the data (Fig. 2). Conditional logistic regression was then used to analyse the association between immune response category and risk of TB disease (Supplementary Table 5). For HLA-DR^+^ CD4^+^ T cells, the risk was greatest for higher responders (OR=1.97, 95% CI=0.86–4.46), and for BCG ELISpot (OR=0.2, 95% CI=0.009–0.91) and CD4^+^ T cells (OR=0.41, 95% CI=0.17–1.01), the risk was lowest for higher responders, confirming the linearity of response. For Ag85A IgG, medium responders were at lowest risk at D0 (OR=0.28, 95% CI=0.0098–0.79) but at D28 the response was linear, with the lowest risk in the higher responders (OR=0.29, 95% CI=0.12–0.72).

**Figure 5:**
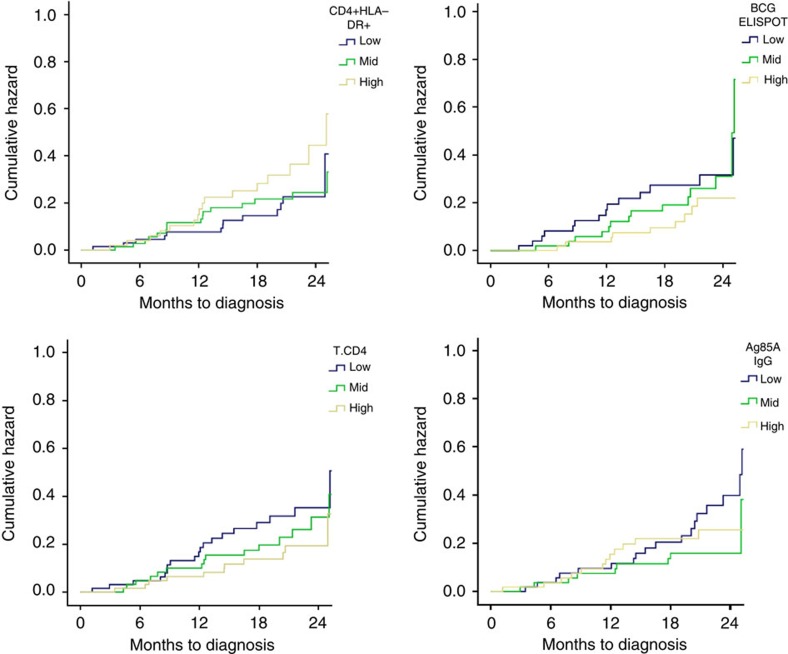
Risk of TB disease over time by magnitude of immune response (TB cases only). Hazard of disease over time for HLA-DR^+^ CD4^+^ T cell, BCG ELISpot, CD4^+^ T-cell and Ag85A IgG OD immune response magnitude is shown for TB case infants. All immune responses were measured at D0 before immunization with MVA85A. For each immune parameter, TB cases were stratified into subgroups divided into thirds according to immune response level (low blue, mid green and high yellow). The plots show the cumulative hazard of TB over time for the three immune level groups.

To determine if there were any time-dependent effects of immune correlates on risk of TB disease, we examined TB disease risk by time for case infants. The risk of TB disease by time for each tertile of immune response category for D0 HLA-DR^+^ CD4^+^ T cells, BCG-specific IFN-γ ELISpot response, CD4^+^ T cells and Ag85A-specific IgG are shown in Fig. 5. The main effect of BCG ELISpot appears to be early in the first 6–12 months of the follow-up period, whereas the effect is more evenly distributed by time for the other immune variables (Fig. 5).

### Ag85A-specific IgG antibody response over time

**Figure 6:**
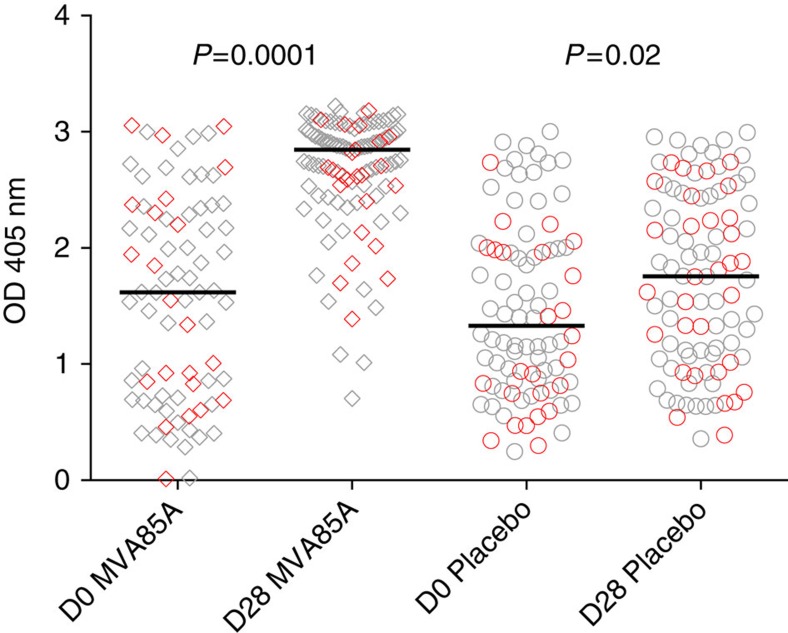
Ag85A IgG is boosted by MVA85A but is also increased in the placebo group from D0 to D28. Ag85A-specific IgG stratified by vaccine group (MVA85A D0 *n*=79, MVA85A D28 *n*=107, placebo D0 *n*=88 and placebo D28 *n*=95). Lines represent median responses; round: placebo, diamond: MVA85A, red: case, grey: control. Although Ag85A IgG is boosted by MVA85A, Ag85A-specific IgG is significantly higher at D28 when compared with D0 in both the placebo and MVA85A groups.

The Mann–Whitney test was used to assess the effect of time and vaccination status on Ag85A-specific IgG for the infant cases and corresponding controls with antibody data at either D0 or D28. The median optical density (OD; 405 nm) at D0 was 1.477 (inter quartile range (IQR)=0.815–2.173) and did not differ by vaccination status (*P*=0.51). MVA85A-vaccinated infants experienced a median increase of 1.226 OD by D28 (*P*<0.0001), whereas control infants experienced a mean increase of 0.425 (*P*=0.02; Fig. 6). The difference between the vaccine and control group at D28 is highly significant (*P*<0.0001). However, the exact quantity of antibody in the MVA85A group is unknown as the upper limit of detection of the ELISA is ∼3 OD and many infants in the MVA85A group were near the upper limit of detection at D28.

## Discussion

In this primary correlates of risk analysis, we have demonstrated that activated HLA-DR^+^ CD4^+^ T cells were associated with subsequent risk of TB disease. This finding was replicated in an independent cohort of *M. tuberculosis*-infected adolescents who either progressed to active TB disease, or who remained healthy over 2 years of follow-up. We have also shown that BCG-specific IFN-γ-secreting T cells measured by ELISpot assay were associated with a reduced risk of TB disease in infants. BCG-specific IFN-γ-secreting T cells were not measured in the adolescent cohort. The effect of BCG-specific IFN-γ-secreting T cells in infants was linear with higher numbers of IFN-γ-secreting T cells associated with a greater reduction in risk of TB disease. The main effect of IFN-γ-secreting T cells appeared to be in the first 6–12 months of follow-up, suggesting an early protective effect of BCG-specific IFN-γ-secreting T cells in infancy. Activated T cells are known to be associated with HIV progression but this is the first report of an association between T-cell activation and TB disease risk^15^,^16^,^17^,^18^. Although BCG immunization has been shown to drive early BCG-specific CD4^+^ T-cell activation in this infant population, we do not know if BCG immunization is driving T-cell activation or if other factors such as immunization with extended programme of immunisation (EPI) vaccines or infection with pathogens is contributing to T-cell activation^19^. Infection with CMV has been shown to drive T-cell activation in HIV-positive patients and we did observe a correlation between T-cell activation and CMV ELISpot response in this study^20^,^21^. In an immune phenotyping study, differences in T-cell activation were largely associated with environmental and not genetic factors, with CMV identified as the major microbial driver of immune variation^22^. The factors that drive variation in BCG efficacy include previous exposure to mycobacteria, age and proximity to the equator^23^; these factors have all been associated with increased T-cell activation^24^,^25^,^26^,^27^,^28^,^29^,^30^. The elevated CD4 T-cell activation observed in *M. tuberculosis*-infected progressors coincides with an elevated type I/II interferon signature, which is suggestive of incipient or sub-clinical TB (Zak *et al*.,^31^ in the press), suggesting a possible link between bacterial replication and T-cell activation. Primary CMV infection typically peaks at birth and again in adolescence^32^,^33^ and may also be contributing to T-cell activation in this adolescent cohort. However, it is likely that there are other, unknown factors driving immune activation in the adolescents, and that these may be different to those driving CD4 T-cell activation in infants. Ultimately, multiple mechanisms of CD4 T-cell activation may be associated with risk of TB.

The cellular immune response to BCG administered at birth in South African infants peaks at 6–10 weeks^19^. The samples collected here were 16–20 weeks post BCG immunization and we see an association between BCG-specific IFN-γ ELISpot response and reduced TB disease risk. The importance of an intact Th1 type cellular immune response in protective immunity has been demonstrated in human genetic studies and murine *M. tuberculosis* challenge experiments^2^,^3^,^4^,^5^,^6^,^8^,^9^, however, this is the first time a role has been demonstrated for magnitude of vaccine-induced IFN-γ-secreting T cells and reduced TB disease risk in a human immune correlates study^11^. A previous study in this infant population found no association of IFN-γ with TB disease risk^11^. Our study differs in several aspects, which could explain the contrasting results including the time point (age) at which the response was measured (at peak or post-peak), IFN-γ assay used (ELISpot versus intracellular cytokine assay), sample type (PBMC versus whole blood), statistical design, more stringent TB case definition and different control definition. In infants, the magnitude of the MVA85A-boosted Ag85A, PPD and BCG-specific IFN-γ ELISpot response is lower than that of UK adults, children or adolescents^12^,^34^,^35^,^36^. It is possible that the lack of efficacy with MVA85A was due to insufficient boosting of a Th1 type immune response in infants.

An unexpected finding was the association of Ag85A-specific IgG measured at D28 (5–7 months of age) with reduced risk of TB. Ag85A-specific IgG increased from D0 to D28 in both treatment groups in this study. Antibody response are likely primed by BCG vaccination at birth. However, they could also be rising due to exposure to environmental mycobacteria. In a cohort of 66 BCG-vaccinated infants in Turkey, PPD-specific IgG levels began to rise at 4 months of age and progressively increased through to the end of the study at 15 months of age^37^. Although there are no published studies describing BCG-specific antibody responses in South African infants, it is possible that Ag85A IgG would have a similar kinetic and would be rising in this infant cohort at the time of randomization.

Although the Ag85A IgG response was boosted after vaccination with MVA85A, there was no significant effect of vaccination status when included as a main effect in our D28 analysis. It is possible that the infant Ag85A antibody response induced by MVA85A was short-lived or that the protective effect of induced antibodies was masked as Ag85A IgG antibodies were also rising in the placebo group in this study. It is also possible that in BCG-vaccinated infants Ag85A IgG is not directly linked with reduced risk but is correlated with an alternative protective immune mechanism induced by BCG. We did not see any significant differences using a MGIA between case and control infants. The lack of difference could be due to sample size or lack of sensitivity of the assay; however, we did not use autologous serum in the MGIA assay and would not therefore have measured any potential effect of antibodies on the control of mycobacterial growth in infant PBMC.

The immune correlates identified in this study would ideally be confirmed in independent clinical studies in BCG-vaccinated infants. However, the infant population in the Western Cape in South Africa has the highest incidence of non-HIV-associated TB recorded in the world at a rate of 1.39 per hundred person years^12^. With such a low incidence rate, the MVA85A trial had to enrol 2,797 infants who were followed up for a median of 24.6 months at a total cost of ∼30 million US dollars to gain sufficient power to determine differences between the placebo and MVA85A arms. The finding that T-cell immunogenicity is reduced in infants compared with adults with MVA85A and other subunit vaccines in development, together with the realization that an adolescent vaccine would have a greater impact on transmission, has led to a refocusing within the field on adolescents as the most important target population^38^. However, the TB incidence rate in adolescents and adults is even lower than in infants and a TB vaccine efficacy trial will therefore be more costly and lengthy in these populations than it was in infants^14^.

Nonetheless, we have identified CD4^+^ T-cell activation as a potential correlate of TB disease risk in BCG-vaccinated infants and have confirmed this finding in an independent adolescent cohort. Moreover, we have identified BCG-specific IFN-γ T cells as a correlate of reduced risk in infants, supporting the concept of Th1 boosting in TB vaccine design. We also identified Ag85A-specific IgG as a potential novel immune correlate of TB disease risk in BCG-vaccinated infants, suggesting a possible role for both Ag85A antibodies and BCG-specific IFN-γ T cells in protection from TB disease. However, these responses may be impaired by T-cell activation and the impact of T-cell activation on disease risk should be considered when designing and testing TB vaccine candidates for these populations.

## Methods

### Case–control design

Infants who were BCG vaccinated within 7 days of birth and enrolled in an efficacy trial of the candidate TB vaccine MVA85A were included in this case–control study, ClinicalTrials.gov number NCT00953927 (Fig. 1). HIV-negative infants testing negative with QuantiFERON TB Gold In-tube test (QuantiFERON), and without known TB exposure were randomized at 4–6 months of age to receive a single intradermal dose of MVA85A or placebo (Candin, a candida skin test antigen)^12^. The trial was approved by the University of Cape Town Faculty of Health Sciences Human Research Ethics Committee, Oxford University Tropical Research Ethics Committee and the Medicines Control Council of South Africa. Informed consent was obtained from the mothers of all infants.

The case definition for TB disease was isolation of *M. tuberculosis* by culture or identification of *M. tuberculosis* by Xpert MTB/RIF (Cepheid) plus evidence of mycobacterial infection defined as two acid-fast-positive smears or QuantiFERON-TB Gold In-tube test conversion from negative to positive or tuberculin skin test ≥15 mm plus radiographic findings compatible with TB and clinical manifestations compatible with TB. Infants who met the primary case definition for TB were included as cases. For each case, three control infants were randomly selected from a pool of control infants. Infants from both the placebo and MVA85A-immunized groups were combined into one control pool for selection. Infants were included in the control pool if they did not convert to latent TB infection based on the QuantiFERON TB Gold In-tube test, had not received TB treatment and had not received TB preventative therapy during study follow-up. Matching was based on gender, ethnic group, Centre for Disease Control (CDC) weight-for-age percentile (±10 points) and time on study (±9 months). Infant PBMC samples stored in liquid nitrogen were available for 55 of 71 TB case infants and for 205 of 213 matched controls (Fig. 1). Viability of thawed PBMC was assessed using flow cytometry with Live/Dead Violet stain (Invitrogen) and phytohaemagglutinin (PHA) was included as a positive control for cell viability on ELISpot plates. For the majority of case infants, controls were from both the placebo and MVA85A arms of the study. However, for eight case infants, the matched controls were all from the placebo arm, and for three placebo infants, the matching controls were all from the MVA85A arm. Controls for whom the case is missing a blood sample are excluded from the analysis.

### Immune response variables

A series of pilot experiments using cellular samples from the MVA85A efficacy trial were performed to select immune response variables for this study^39^. Infants selected for these pilot studies were non-case, non-control infants receiving either MVA85A or placebo^39^. Results are described by Harris *et al*., and methods are summarized in Supplementary Table 6 (ref. 39). As cell number was limited and the magnitude of the antigen-specific immune response was low^12^,^39^, we selected assays that measured different components of the immune response, and those assays which did not correlate with each other. Immune assays that were initially reviewed for inclusion in this correlates analysis and ultimately selected are listed in Supplementary Tables 6 and 7.

ELISpot responses were log10-transformed and where the response was zero, a value of 0.3 log10 was used. For analysis of IFN-γ, samples were only included if PHA≥1,000 SFCs per million PBMCs. For all other analyses, samples were included if the percentage of live T cells was ≥50% or if flow cytometry was not performed on that sample then PHA≥1,000 SFCs per million PBMCs. Ag85A-specific IgG in the plasma was measured as an exploratory analysis by ELISA. The laboratory staff performing the assays were blinded to case–control and vaccine group status of infant samples.

### *Ex vivo* IFN-γ ELISpot assay

The *ex vivo* IFN-γ ELISpot assay was performed using a human IFN-γ ELISpot kit (capture mAb -D1K; Mabtech). Duplicate wells containing 3 × 10^5^ PBMC were stimulated for 18 h with antigen, PHA as a positive control or media alone as a negative control. Antigens were: a single pool of Ag85A peptides, consisting of 66 15-mer peptides, overlapping by 10 amino acids (2 μg ml^−1^ per peptide; Peptide Protein Research); BCG from pooled SSI vaccine vials (2 × 10^5^ colony-forming units (CFUs) per ml); PPD from *M. tuberculosis* (20 μg ml^−1^; Statens Serum Institute (SSI)); pools of peptides with knows CD8 T-cell epitopes from flu, Epstein–Barr virus (EBV) and CMV peptides (10 μg ml^−1^ per peptide; Peptide Protein Research); and combined TB10.3 and TB10.4 peptides (10 μg ml^−1^ per peptide; Peptide Protein Research). Results are reported as SFCs per million PBMCs, calculated by subtracting the mean of the unstimulated wells from the mean of antigen wells and correcting for the numbers of PBMC in the wells. A response was considered positive if the mean number of spots in the antigen well was at least twice the mean of the unstimulated wells and at least five spots greater.

### Cell surface flow cytometry

PBMC were washed and stained with 5 μl Live/Dead Violet (Invitrogen) followed by surface staining with the following titrated antibodies: 0.5 μl CD3-AF700 (clone UCHT1, Ebioscience), 2 μl CD4-APC (clone RPA-T4, BioLegend), 2 μl CD8-Efluor605 (clone RPA-T8, Ebioscience), 2 μl CD14-PE/Cy7 (clone HCD14, BioLegend), 2 μl CD16-AF488 (clone 3G8, BioLegend), 1 μl CD19-PE/Cy5 (clone HIB19, BioLegend), 2 μl CD25-APC/Cy7 (clone BC96, BioLegend), 2 μl CD127-NC650 (clone eBioRDR5, Ebioscience), 15 μl HLA-DR-PE (clone L243 BioLegend). Fluorescence minus one controls were used to set gates for CD25, CD127 and HLA-DR. Samples were acquired on a BD LSR II flow cytometer. Results are presented as percentages of cells after gating out of dead cells and doublets. CD4^+^ and CD8^+^ T cells were identified as CD3^+^ cells, whereas CD14^+/−^ and CD16^+/−^ cells were identified as CD3^−^ and CD19^−^ populations. CD25^+^ CD27^−^ populations were gated on the CD4^+^ cells.

### Mycobacterial growth inhibition assay

Duplicate 2-ml screw-cap tubes containing 1 × 10^6^ PBMCs in 600 μl of medium were rotated at 37 °C with ∼600 CFUs of BCG Pasteur stock for 4 days. The PBMCs were then lysed with sterile water, and the lysate transferred to a Bactec MGIT supplemented with PANTA antibiotics and OADC enrichment broth. The tube was placed in a Bactec MGIT 960 and incubated until growth was detected (time to positivity).

### Ag85A IgG ELISA

Nunc immunoplates were coated overnight at 4 °C with 50 μl sodium carbonate buffer containing 1.5 μg ml^−1^ recombinant antigen 85A protein. Plates and reagents were brought to room temperature and the plate washed three times with PBS, 5% Tween-20 (v/v). Plates were blocked for 1 h at room temperature with 100 μl blocking buffer (PBS, 5% Milk Blocking (w/v). All serum samples were diluted 1/100 with PBS, 5% Milk Blocking (w/v) and 50 μl of each diluted sample was plated in triplicate. Controls and plate blanks consisting of assay diluent alone with no serum were added in triplicate. Plates were sealed and incubated for 2 h at room temperature. After washing 50 μl of a 1:500 dilution of goat anti-human (KPL) detection antibody was added to each well for 1 h at room temperature. For colour development, 50 μl TMB (BD) was added to each well. Plates were incubated for 15 min in the dark before the reaction was stopped by adding 50 μl of 2 M Sulfuric Acid (Sigma) to each well. Absorbance was measured using a microplate reader at 450 nm.

### Statistical analysis

Prior to data analysis, a statistical analysis plan was prepared in which primary and secondary analyses were defined and the methods of analysis were specified (available on request). Briefly, 22 primary immune variables were selected for inclusion in the case–control study (Supplementary Table 7). For each variable, a conditional logistic regression analysis was used to assess the association between D0 immune response and risk of TB disease. To adjust for multiple comparisons, the FDR was calculated for each variable using the method of Benjamini and Hochberg^13^. FDR values less than or equal to 0.2 were considered significant. To assess the predictive value of an immune variable, the AUROC curve was computed. Immune variables with AUROC values above 0.80 were considered good predictors of TB disease risk.

Secondary analyses included conditional logistic regression analysis of D28 immune responses for each of the primary variables. Regression models with and without vaccination status (MVA85A or placebo) included as a main effect were examined. In addition to the primary variables, D0 and D28 Ag85A-specific IgG antibody levels were analysed by conditional logistic regression.

Immune response variables that were significant at D0 or D28, were categorized as low, medium or high based on tertiles and conditional logistic regression was performed to assess linearity. To explore any time-dependent effect of immune correlates, plots of the cumulative hazard function (Breslow estimator) for immune response variables (by tertile of immune response) were plotted for case infants only.

The change in antibody from D0 to D28 was assessed using generalized estimating equations as described in Yang and Tsiatis^40^. For our setting, the cluster variable was limited to participant ID and ignored the matching. However, only controls for whom the case had data were included. We also used an unstructured covariance matrix to describe the variance in antibody titres over time.

Outliers as defined by Tukey's rule were excluded from the primary analysis. Outliers were identified via a review of the distribution of the variables. Tukey's rule, which provides a distribution-free method for identification of outliers, was be used to set upper and lower limits for preliminary review and identification of outliers. After determining the 25th and 75th percentiles, the upper limit is set as P75+3 times the interquartile range, or P75-P25. Likewise, the lower limit is P25—3(P75-P25). Following this review, only outliers which were identified and determined to be the result of contamination, operator error or mechanical failure were excluded from the primary analyses. For the day 0 primary analysis, out of 3,918 total measurements across 22 immune variables, 19 data points were identified as statistical outliers (Supplementary Table 8). Following investigation of the raw data, 12 out of 19 data points were retained and 7 data points excluded. Exclusions were due to high background in the ELISpot assay or PHA response <1,000 SFCs per million PBMCs.

### The ACS

We used samples from the ACS for validation of the finding that elevated HLA-DR^+^ CD4^+^ T cells correlated with risk of TB. The ACS was a prospective epidemiological cohort study of *M. tuberculosis* infection and TB disease in adolescents, aged 12–18 years, from the Western Cape of South Africa^14^. A total of 6,363 adolescents were recruited at high schools and followed for 2 years. Latent *M. tuberculosis* infection was diagnosed by tuberculin skin test or QuantiFERON Gold in Tube assay and PBMCs were collected at enrolment and end of study (24 months) in all, and in an active follow-up group also at 6, 12 and 18 months^31^. Participants suspected of having TB were investigated through sputum smears, culture and chest X-ray. A protocol-defined incident case of TB was any case confirmed by two or more sputum smears positive for acid-fast bacilli and/or one positive sputum culture for *M. tuberculosis*. After excluding individuals with a previous history of TB disease, we examined the frequency of HLA-DR-expressing CD4^+^ T cells in 61 progressor samples (from 30 progressor adolescents) and 132 control samples (from 59 matched control adolescents). All available PBMC samples from progressors and matched controls were included in the analysis. Controls were matched on age, gender, ethnicity and high school. HLA-DR-expressing CD4^+^ T cells were measured in PBMC by first gating on singlet cells, then selecting cells with consistent fluorescence during acquisition (time gate), followed by a side scatter/CD45 gate to select lymphocytes. Thereafter CD14^−^ CD3^+^ T cells and then CD4^+^ CD8^−^ cells were selected. Frequencies of HLA-DR-expressing CD4^+^ T cells are reported as a proportion of total live CD4^+^ T cells. Data were analysed using conditional logistic regression as described above comparing progressors to matched controls.

## Additional information

**How to cite this article:** Fletcher, H. A. *et al*. T cell activation is an immune correlate of risk in BCG vaccinated infants. *Nat. Commun.* 7:11290 doi: 10.1038/ncomms11290 (2016).

## Supplementary Material

Supplementary Information Supplementary Figure 1-2, Supplementary Tables 1-8

## Figures and Tables

**Table 1:** Conditional logistic regression.

Quantitative variable	*N*	Cases	Est OR*	95% CI	*P* value	FDR value	AUROC
*Estimated OR of TB disease from a conditional logistic regression of day 0 immunological variable*							
CD3^+^ T cell	186	50	0.98	(0.95, 1.02)	0.394	0.656	0.542
CD4^+^ T cell	186	50	0.95	(0.92, 0.99)	0.027	0.198	0.592
CD4^+^HLADR^+^ T cell	186	50	1.12	(1.04, 1.2)	0.002	0.047	0.619
CD4^+^CD25^+^CD127^−^	186	50	0.82	(0.54, 1.25)	0.356	0.647	0.550
CD8^+^ T cell	186	50	1.04	(0.99, 1.09)	0.159	0.399	0.556
HLA-DR^+^ CD8^+^ T cell	186	50	1.02	(1.00, 1.04)	0.056	0.279	0.579
CD14^+^CD16^+^ e	186	50	1.00	(0.80, 1.25)	0.994	0.994	0.448
CD14^+^CD16^−^	186	50	1.01	(0.93, 1.09)	0.857	0.994	0.505
CD19^+^ B cell	186	50	1.04	(0.99, 1.09)	0.144	0.399	0.580
BCG MGIA	80	30	0.31	(0.03, 3.08)	0.318	0.585	0.529
85A ELISpot	125	40	NA	NA	NA	NA	NA
BCG ELISpot	138	43	0.46	(0.23, 0.93)	0.03	0.198	0.591
PPD ELISpot	90	32	0.72	(0.3, 1.7)	0.453	0.639	0.586
TB10.3/10.4 ELISpot	90	32	0.40	(0.13, 1.31)	0.131	0.314	0.567
EBV ELISpot	100	34	0.98	(0.34, 2.82)	0.966	0.994	0.5
CMV ELISpot	126	40	0.87	(0.54, 1.41)	0.582	0.776	0.52
FLU ELISpot	100	34	NA	NA	NA	NA	NA
GAM.DEL (putative)	186	50	1.41	(0.93, 2.12)	0.104	0.399	0.561
NK.16NEG (putative)	186	50	0.92	(0.79, 1.08)	0.331	0.647	0.541
NK.16POS (putative)	186	50	0.99	(0.89, 1.1)	0.851	0.994	0.507
CD14^+^CD16^+^ /CD3^+^	186	50	1.37	(0.05,38.72)	0.854	0.994	0.515
CD14^+^CD16^−^/CD3^+^	186	50	0.96	(0, 13609)	0.993	0.994	0.442
							
*Exploratory variable*							
Ag85A IgG	145	46	0.70	(0.46, 1.08)	0.106		0.589
							
*Estimated OR of TB disease from a conditional logistic regression of day 28 immunological variable*							
CD3^+^ T cell	200	52	0.99	(0.96, 1.03)	0.746	0.871	0.511
CD4^+^ T cell	200	52	0.98	(0.94, 1.02)	0.344	0.871	0.526
HLA-DR^+^ CD4^+^ T cell	200	52	1.12	(1.05, 1.19)	0.001	0.013	0.653
CD4^+^CD25^+^CD127^−^	200	52	0.68	(0.37, 1.25)	0.281	0.871	0.528
CD8^+^ T cell	200	52	1.02	(0.96, 1.08)	0.423	0.871	0.555
HLA-DR^+^ CD8^+^ T cell	200	52	1.02	(1.00, 1.04)	0.031	0.346	0.604
CD14^+^CD16^+^	200	52	0.92	(0.74, 1.15)	0.516	0.871	0.534
CD14^+^CD16^−^	200	52	0.99	(0.92, 1.07)	0.752	0.871	0.502
CD19^+^ B cell	200	52	1.01	(0.96, 1.07)	0.610	0.871	0.538
BCG MGIA	74	29	0.49	(0.05, 4.60)	0.458	0.871	0.548
85A ELISpot	165	49	0.69	(0.4, 1.19)	0.183	0.871	0.552
BCG ELISpot	165	49	0.70	(0.4, 1.21)	0.202	0.871	0.557
PPD ELISpot	146	46	0.56	(0.31, 1.01)	0.055	0.404	0.575
TB10.3/10.4 ELISpot	146	46	0.99	(0.4, 2.47)	0.990	1	0.499
EBV ELISpot	155	48	1.20	(0.49, 2.96)	0.688	0.871	0.507
CMV ELISpot	163	49	1.08	(0.72, 1.61)	0.723	0.871	0.523
FLU ELISpot	154	48	1.00	(0.11. 8.87)	1	1	0.494
GAM.DEL (putative)	200	52	0.98	(0.74, 1.31)	0.866	0.953	0.508
NK.16NEG (putative)	200	52	1.08	(0.94, 1.23)	0.245	0.871	0.527
NK.16POS (putative)	200	52	0.96	(0.85, 1.08)	0.464	0.871	0.532
CD14^+^CD16^+^/CD3^+^	200	52	0.03	(0, 923.33)	0.608	0.871	0.494
CD14^+^CD16^−^/CD3^+^	200	52	0.45	(0.02, 9.45)	0.497	0.871	0.529
							
*Exploratory variable*							
Ag85A IgG	188	56	0.62	(0.41, 0.93)	0.019		0.615

AUROC, area under the receiver operating characteristic; CI, confidence interval; FDR, false discovery rate; NA, model did not converge; results not available; Est OR, estimated odds ratio.

^*^Represents the odds ratio for a 1 unit change in the immune response.

**Table 2:** Conditional logistic regression of HLA-DR^+^ CD4^+^ T cells in the Adolescent Cohort Study.

Time to TB diagnosis	*N*	Progressors	OR	95% CI	*P* value
Any	193	61	1.387	1.068, 1.801	0.014
All controls and progressors <18 months	173	50	1.402	1.067, 1.843	0.015
All controls and progressors <12 months	140	34	1.537	1.106, 2.135	0.01
All controls and progressors <6 months	118	16	1.501	1.047, 2.152	0.027

CI, confidence interval; OR, odds ratio; TB, tuberculosis.
