# Corrigendum: T-cell activation is an immune correlate of risk in BCG vaccinated infants

**DOI:** 10.1038/ncomms11633

**Published:** 2016-05-06

**Authors:** Helen A. Fletcher, Margaret A. Snowden, Bernard Landry, Wasima Rida, Iman Satti, Stephanie A. Harris, Magali Matsumiya, Rachel Tanner, Matthew K. O'Shea, Veerabadran Dheenadhayalan, Leah Bogardus, Lisa Stockdale, Leanne Marsay, Agnieszka Chomka, Rachel Harrington-Kandt, Zita-Rose Manjaly-Thomas, Vivek Naranbhai, Elena Stylianou, Fatoumatta Darboe, Adam Penn-Nicholson, Elisa Nemes, Mark Hatherill, Gregory Hussey, Hassan Mahomed, Michele Tameris, J. Bruce McClain, Thomas G. Evans, Willem A. Hanekom, Thomas J. Scriba, Helen McShane

*Nature Communications* 7, Article number: 11290 10.1038/ncomms11290 (2016); Published: 04122016; Updated: 05062016

The original version of this Article contained an error in the spelling of the author Mark Hatherill, which was incorrectly given as Mark Hatheril. This has now been corrected in both the PDF and HTML versions of the Article.

