# Supplementary material for: T-cell activation is an immune correlate of risk in BCG vaccinated infants
Source: Nat Commun. 2016 Apr 12;7:11290. doi: 10.1038/ncomms11290 (PMC4832066; doi:10.1038/ncomms11290)
Supplement: Supplementary Information — Supplementary Figure 1-2, Supplementary Tables 1-8 [file ncomms11290-s1.pdf]

Supplementary Figure 1

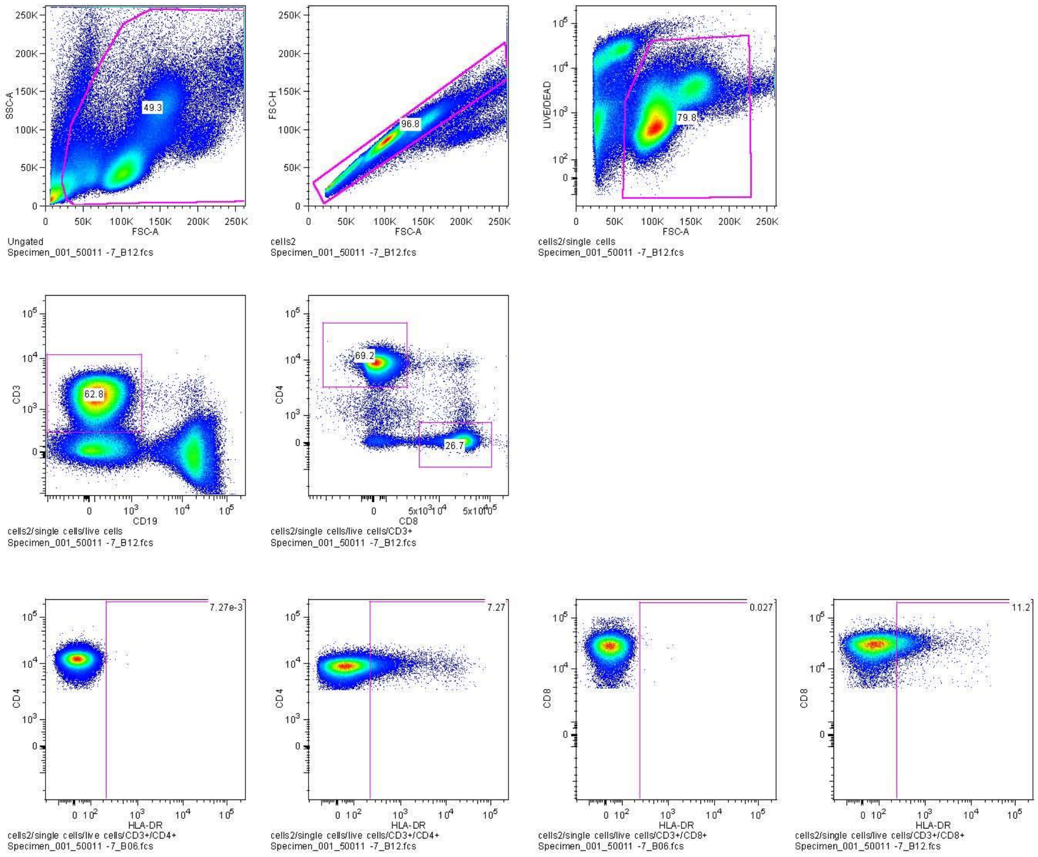

FMO control
(no HLA-DR antibody)

FMO control
(no HLA-DR antibody)

Supplementary Figure 1. Gating strategy for HLA-DR+ T-cells.

A gate was drawn around lymphocytes based on cell size (FSC-A/SSC-A) and then a second gate was drawn around single cells (FSC-A/FSC-H). Live singlet cells were then gated as CD3+CD19. CD3+ T-cells were then individually gated as either CD4+ or CD8+. A fluorescence minus one control tube (FMO) containing all stains except HLA-DR was used to determine the position of the HLA-DR+ gate on either CD4+ or CD8+ T-cells.

Supplementary Figure 2

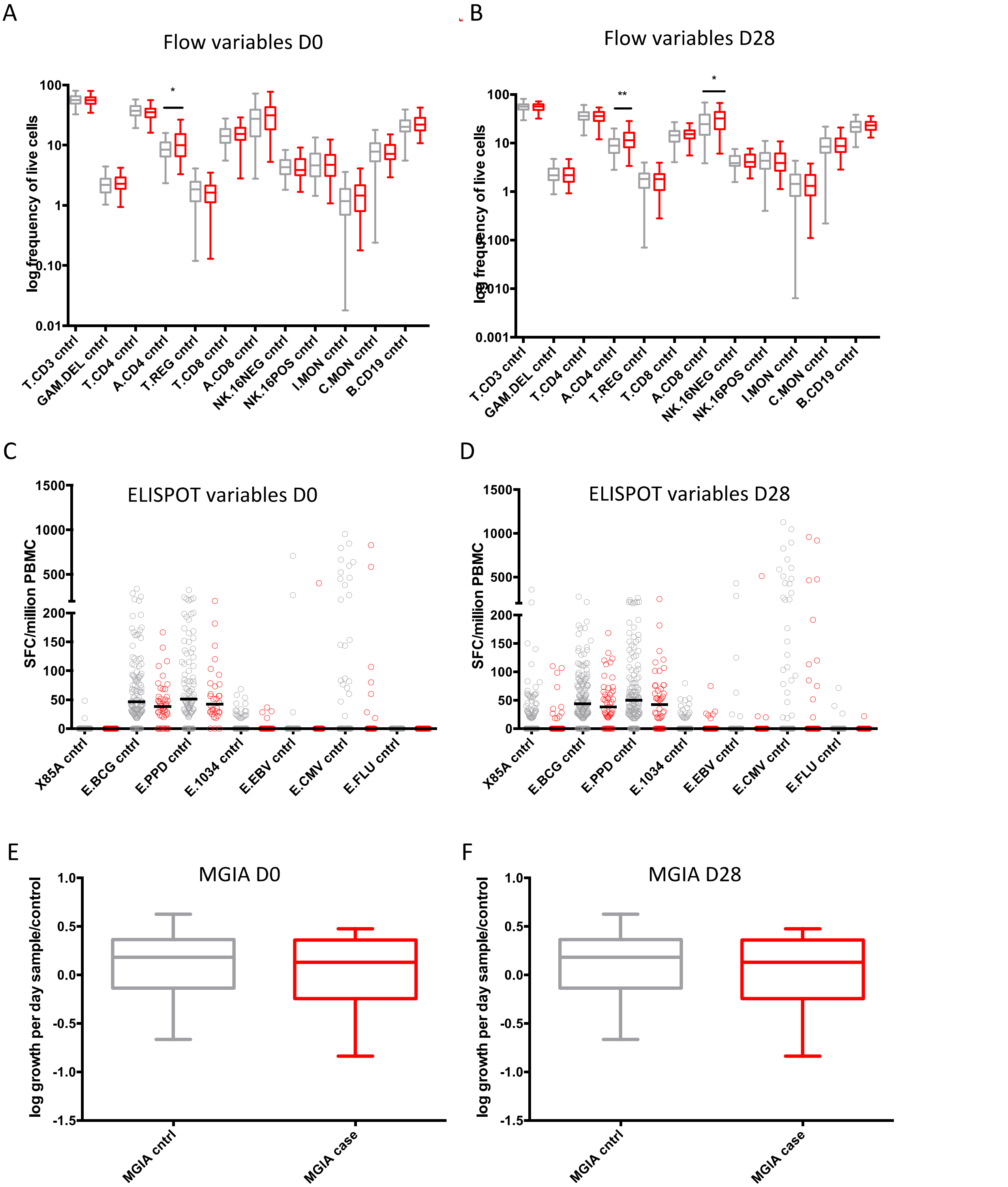

Supplementary Figure 2. Distribution of immune response variables in case and control infants at D0 and D28.

Infants are stratified according to TB case (red) or control (grey) status. Immune responses at D0 (panels A, C, E) or D28 (panels B, D, F) are shown. The log frequency of live cells expressing cell surface phenotype markers is shown. SFC/million PBMC is the number of IFN-γ spot forming cells per million PBMC (only results from infants with a PHA response greater than 1000 SFC/million are shown).

Supplementary Table 1. Spearman rank correlations between activated T cells and CMV response

D0 Correlations					
		HLA-DR+ CD4+	CD4+	CD8+HLADR+	ELISpot CMV
HLA-DR+ CD4+	R value	1.000	**-.427**	**.530**	.050
	P value	.	**.000**	**.000**	.529
	N	192	**192**	**192**	163
CD4+	R value	**-.427**	1.000	**-.391**	-.074
	P value	**.000**	.	**.000**	.348
	N	**192**	**192**	**192**	163
CD8+HLADR+	R value	**.530**	**-.391**	1.000	**.301**
	P value	**.000**	**.000**	.	**.000**
	N	**192**	**192**	192	**163**
CMV ELISpot	R value	.050	-.074	**.301**	1.000
	P value	.529	.348	**.000**	.
	N	163	163	**163**	180
**D28 Correlations**					
HLA-DR+ CD4+	R value	1.000	**-.280**	**.556**	.116
	P value	.	**.000**	**.000**	.115
	N	209	**209**	**209**	187
CD4+	R value	**-.280**	1.000	**-.316**	-.014
	P value	**.000**	.	**.000**	.849
	N	**209**	209	**209**	187
CD8+HLADR+	R value	**.556**	**-.316**	1.000	**.222**
	P value	**.000**	**.000**	.	**.002**
	N	**209**	**209**	209	**187**
CMV ELISpot	R value	.116	-.014	**.222**	1.000
	P value	.115	.849	**.002**	.
	N	187	187	**187**	209

Significant correlations highlighted in bold

Supplementary Table 2. Estimated odds ratio for the association between risk of TB disease and HLA-DR+ CD4+ by treatment group MVA85A or Placebo

**Estimated odds ratio for the association between risk of TB disease and A.CD4 by treatment group MVA85A or Placebo**							
**Day**	Model	N	Cases	Est OR	LL	UL	P value
**D0**	CD4+ HLA-DR	186	50	1.12	1.04	1.21	0.002
**D0**	CD4+ HLA-DR **+** MVA85A or Placebo	186	50	1.12	1.04	1.21	0.002
**D0**	CD4+ HLA-DR *MVA85A or placebo^	186	50	1.15	1.02	1.29	0.024
**D28**	CD4+ HLA-DR	200	52	1.12	1.05	1.19	<0.001
**D28**	CD4+ HLA-DR + MVA85A or placebo	200	52	1.12	1.05	1.19	<0.001
**D28**	CD4+ HLA-DR * MVA85A or placebo ^	200	52	1.09	1.01	1.18	0.030
**^Est OR is for the control group.**							

Supplementary Table 3. **Estimated Odds-Ratio (OR) of TB disease from a Conditional Logistic Regression of Day 28 immunological variable for MVA85A and placebo arms**

**Quantitative variable**	Est OR* MVA85A	95% CI MVA85A	P value MVA85A	Est OR* Placebo	95% CI Placebo	P value Placebo
**CD3+ T cell**	1.001	0.941, 1.065	0.97	0.959	0.895, 1.028	0.236
**CD4+ T cell**	0.995	0.929, 1.066	0.887	0.936	0.856, 1.023	0.144
**CD4+HLADR+ T cell**	1.16	0.993, 1.356	0.061	1.075	0.956, 1.21	0.227
**CD4+CD25+CD127- T cell**	0.905	0.415, 1.971	0.801	0.895	0.392, 2.041	0.792
**CD8+ T cell**	0.999	0.893, 1.117	0.988	1.017	0.924, 1.12	0.73
**HLA-DR+ CD8+ T cell**	1.016	0.983, 1.051	0.345	1.008	0.978, 1.04	0.594
**CD14+CD16+ monocyte**	0.784	0.497, 1.237	0.296	0.939	0.596, 1.48	0.786
**CD14+CD16- monocyte**	1.002	0.861, 1.167	0.975	1.038	0.896, 1.202	0.621
**CD19+ B cell**	1.012	0.93, 1.101	0.787	1.057	0.964, 1.159	0.241
**BCG MGIA**	4.331	0.002, 10654.906	0.713	1.373	0.01, 187.859	0.9
**85A ELISpot**	0.671	0.286, 1.576	0.36	76.576	0, 1360699283.119	0.61

**BCG ELISpot**	1.139	0.488, 2.659	0.764	0.574	0.189, 1.738	0.326
**PPD ELISpot**	0.559	0.212, 1.474	0.24	0.225	0.033, 1.53	0.127
**TB10.3/10.4 ELISpot**	0.49	0.063, 3.837	0.497	0.47	0.104, 2.117	0.326
**EBV ELISpot**	1.948	0.377, 10.062	0.426	0.662	0.112, 3.923	0.649
**CMV ELISpot**	0.844	0.427, 1.671	0.627	1.476	0.598, 3.64	0.398
**FLU ELISpot**	56.51	0, 311888994.246	0.61	0.687	0.351, 1.347	0.275
**GAM.DEL (putative)**	1.413	0.669, 2.987	0.365	1.128	0.847, 1.502	0.412
**NK.16NEG (putative)**	1.449	0.904, 2.321	0.123	0.981	0.751, 1.281	0.888
**NK.16POS (putative)**	0.829	0.624, 1.102	0.197	5.372	0.014, 2069.112	0.58
**CD14+CD16+ /CD3+**	1.059	0.002, 480.384	0.985	0.139	0, 533725349.394	0.861
**CD14+CD16- /CD3+**	0	0, 31198.833	0.32	0.516	0.212, 1.256	0.145
**Ag85A IgG**	0.749	0.25, 2.24	0.605	0.959	0.895, 1.028	0.236

Supplementary Table 4. Conditional Logistic Regression with Z-transformed variables

**Estimated Odds-Ratio (OR) of TB disease from a Conditional Logistic Regression of Day 0 immunological variable**							
**Quantitative variable**	N	Cases	Est OR*	95% CI	P value	FDR value	AUROC
**CD3+ T cell**	186	50	0.86	0.61, 1.22	0.397	0.69	-
**CD4+ T cell**	186	50	0.651	0.45, 0.95	0.025	0.18	0.583
**CD4+HLADR+ T cell**	186	50	1.828	1.25, 2.68	0.002	0.04	0.618
**CD4+CD25+CD127- T cell**	186	50	0.85	0.59, 1.23	0.385	0.69	-
**CD8+ T cell**	186	50	1.236	0.93, 1.64	0.142	0.36	-
**HLA-DR+ CD8+ T cell**	186	50	1.349	1, 1.82	0.05	0.26	-
**CD14+CD16+ monocyte**	186	50	1.021	0.75, 1.39	0.894	0.89	-
**CD14+CD16- monocyte**	186	50	1.029	0.73, 1.46	0.873	0.89	-
**CD19+ B cell**	186	50	1.267	0.93, 1.74	0.141	0.36	-
**BCG MGIA**	80	30	-	-	-	-	-
**85A ELISpot**	125	40	0.648	0.012, 35.35	0.832	0.89	-
**BCG ELISpot**	138	43	0.502	0.29, 0.86	0.013	0.14	0.575
**PPD ELISpot**	90	32	0.608	0.36, 1.03	0.064	0.27	-
**TB10.3/10.4 ELISpot**	90	32	0.666	0.38, 1.17	0.154	0.36	-

**EBV ELISpot**	100	34	0.959	0.73, 1.25	0.758	0.89	-
**CMV ELISpot**	126	40	0.92	0.61, 1.39	0.69	0.89	-
**FLU ELISpot**	100	34	5.962	0.001, 35600.91	0.687	0.89	-
**GAM.DEL (putative)**	186	50	1.418	0.95, 2.12	0.09	0.32	-
**NK.16NEG (putative)**	186	50	0.728	0.41, 1.31	0.289	0.61	-
**NK.16POS (putative)**	186	50	0.973	0.72, 1.32	0.857	0.89	-
**CD14+CD16+ /CD3+**	186	50	1.021	0.76, 1.38	0.892	0.89	-
**CD14+CD16- /CD3+**	186	50	1.03	0.73, 1.45	0.867	0.89	-
**Exploratory Variable**							
**Ag85A IgG**	145	46	0.753	0.54, 1.05	0.095	-	0.563
**Estimated Odds-Ratio (OR) of TB disease from a Conditional Logistic Regression of Day 28 immunological variable**							
**CD3+ T cell**	200	52	0.928	0.65, 1.32	0.681	0.88	-
**CD4+ T cell**	200	52	0.818	0.58, 1.17	0.265	0.83	0.522
**HLA-DR+ CD4+ T cell**	200	52	1.795	1.29, 2.49	0	0.00	0.643
**CD4+CD25+CD127- T cell**	200	52	0.824	0.57, 1.2	0.317	0.83	-
**CD8+ T cell**	200	52	1.153	0.85, 1.56	0.369	0.86	-
**HLA-DR+ CD8+ T cell**	200	52	1.398	1.04, 1.87	0.025	0.26	-
**CD14+CD16+ monocyte**	200	52	0.908	0.67, 1.23	0.533	0.88	-
**CD14+CD16-**	200	52	0.96	0.71, 1.30	0.794	0.92	-

**monocyte**							
**CD19+ B cell**	200	52	1.097	0.79, 1.52	0.574	0.88	-
**BCG MGIA**	74	29	-	-	-		-
**85A ELISpot**	165	49	0.852	0.64, 1.14	0.279	0.83	-
**BCG ELISpot**	165	49	0.826	0.57, 1.19	0.306	0.83	0.573
**PPD ELISpot**	146	46	0.765	0.54, 1.09	0.142	0.83	-
**TB10.3/10.4 ELISpot**	146	46	0.975	0.7, 1.36	0.88	0.92	-
**EBV ELISpot**	155	48	1.063	0.79, 1.43	0.686	0.88	-
**CMV ELISpot**	163	49	1.005	0.77, 1.32	0.969	0.97	-
**FLU ELISpot**	154	48	0.947	0.71, 1.26	0.712	0.88	-
**GAM.DEL (putative)**	200	52	0.976	0.73, 1.3	0.87	0.92	-
**NK.16NEG (putative)**	200	52	1.352	0.84, 2.17	0.21	0.83	-
**NK.16POS (putative)**	200	52	0.872	0.62, 1.24	0.442	0.88	-
**CD14+CD16+ /CD3+**	200	52	0.902	0.66, 1.24	0.524	0.88	-
**CD14+CD16- /CD3+**	200	52	0.931	0.69, 1.27	0.651	0.88	-
**Exploratory Variable**							
**Ag85A IgG**	188	56	0.738	0.54, 1.01	0.057		0.598
**NA = model did not converge; results not available**<br>***represents the odds ratio for a 1 unit change in the immune response**							

Supplementary Table 5. Conditional Logistic Regression based on tertile of immune response

**Conditional Logistic Regression based on tertile of immune response**										
	**D0**					**D28**				
	Range	N	OR	95% CI	p	Range	N	OR	95% CI	p
**HLADR+ CD4+ T-cells**										
Low	2.34, 7.19	62	Ref	-	**0.26**	2.79, 7.31	67	-	-	**0.011**
Medium	7.20, 10.53	62	1.28	0.59, 2.80	0.53	7.32, 11.40	67	1.28	0.49, 3.37	0.61
High	10.54, 26.70	62	1.97	0.86, 4.46	0.11	11.41, 39.90	66	3.16	1.36, 7.37	0.008
**BCG ELISpot**										
Low	0.30, 1.50	47	Ref	-	**0.081**	0.30, 1.45	59	-	-	**0.38**
Medium	1.51, 1.81	46	0.93	0.37, 2.36	0.88	1.46, 1.77	51	0.6	0.24, 1.47	0.26
High	1.82, 2.53	45	0.28	0.09, 0.91	0.034	1.78, 2.49	55	0.61	0.27, 1.37	0.23
**CD4+ T-cells**										
Low	0.01, 1.01	48	Ref	-	**0.15**	0.36, 1.98	63	Ref	-	**0.042**
Medium	1.02, 1.98	49	0.68	0.30, 1.53	0.35	1.99, 2.72	62	0.35	0.15, 0.81	0.014
High	1.99, 3.06	48	0.41	0.17, 1.01	0.051	2.73, 3.22	63	0.66	0.27, 1.63	0.36
**Ag85A-specific IgG**										
Low	0.01, 1.01	48	Ref	-	**0.031**	0.36, 1.98	63	Ref	-	**0.026**
Medium	1.02, 1.98	49	0.28	0.098, 0.79	0.016	1.99, 2.72	62	0.73	0.34, 1.54	0.4
High	1.99, 3.06	48	0.44	0.19, 1.042	0.062	2.73, 3.22	63	0.29	0.12, 0.72	0.007

Table 6: Assay identification for C-020-485 case-control correlates analysis: Preliminary selection

Study Group / Pilot Study	Sample type	Assay	Stimulation condition	Reliability (externally validated assay or ICC)	Sample available	Detection of antigen specific immune response	Correlation between assays (Spearmans correlation p<0.01)
Group 2	Fresh PBMC	ELISPOT	UNS, BCG, Ag85A peptides	externally validated	No	Yes	ELISPOT/WB-ICS
Group 3	Fresh PBMC	PBMC ICS	UNS, BCG, Ag85A peptides	externally validated	No	Yes	
Group 4	Whole blood	Whole blood ICS	UNS, BCG, Ag85A peptides	externally validated	No	Yes	ELISPOT/WB-ICS
Pilot 1	Frozen PBMC	Assay 1: ELISPOT (2 antigens)	UNS, BCG, Ag85A peptides	passed	Yes	Yes	ELISPOT/ GEX Ag85A peptides
Pilot 1	Frozen PBMC	Assay 2: Illumina HT-12 Gene expression analysis (GEX) (5 conditions)	UNS, BCG, Ag85A peptides	passed	Yes	Yes	ELISPOT/ GEX Ag85A peptides
Pilot 1	Frozen PBMC	Assay 3: Mycobacterial growth inhibition assay (MGIA) (1 mycobacterial strain)	BCG Pasteur	passed	Yes	-	-
Pilot 1	Frozen PBMC	Assay 4: Flow surface staining Live/dead, CD3, CD4, CD8, γδ, CD19, CD14 (6 populations)	UNS	passed	Yes	-	-
Pilot 1	Supernatant from stimulated frozen PBMC	Assay 5: Luminex (42 plex) MGIA supernatants<br><br>Assay 6: Luminex (42 plex) GEX supernatants	UNS, BCG, Ag85A peptides	Externally validated	Yes	Yes	Some subsets of analytes and GEX/MGIA NB. Supernatants collected from MGIA and GEX experiments
Pilot 2	Frozen PBMC	ELISPOT (duplicate wells) (3 antigens)	UNS, BCG, PPD, Ag85A peptides	passed	Yes	Yes	ELISPOT/ GEX Ag85A peptides/ Ki67 proliferation
Pilot 2	Frozen PBMC	ELISPOT (triplicate wells) (3 antigens)	UNS, BCG, PPD, Ag85A peptides	passed	Yes	Yes	ELISPOT/ GEX Ag85A peptides/ Ki67 proliferation
Pilot 2	Frozen PBMC	Illumina HT-12 Gene expression analysis (GEX) (5 conditions)	UNS, BCG, Ag85A peptides	passed	Yes	Yes	ELISPOT/ GEX Ag85A peptides
Pilot 2	Frozen PBMC	Mycobacterial growth inhibition assay (MGIA) (2 strains of mycobacteria)	BCG Pasteur, MTB H37rV	passed	Yes	-	MGIA and CD14+CD16+ monocytes on cell surface flow
Pilot 2	Frozen PBMC	Flow surface staining Live/dead, CD3,	UNS	passed	Yes	-	MGIA and CD14+CD16+ monocytes on

		CD4, CD8, γδ, CD19, CD14, CD16, CD25, CD127, CTLA4 (90 cell populations)					cell surface flow
Pilot 2	Frozen PBMC	Assay 7: Ki67 proliferation assay (4 parameters – 2 antigens CD4 and CD8)	UNS, PPD, Ag85A peptides	Not done	Yes	Yes	ELISPOT/ Ki67 proliferation
Pilot 3	Frozen PBMC (short rest)	ELISPOT (8 antigens)	UNS, BCG, PPD, Ag85A peptides, Flu+CMV+EBV peptides, tetanus toxoid, TB10.3/4	passed	Yes	Yes	-
Pilot 3	Frozen PBMC (long rest)	ELISPOT (8 antigens)	UNS, BCG, PPD, Ag85A peptides, Flu+CMV+EBV peptides, tetanus toxoid, TB10.3/4	passed	Yes	Yes	-

The maximum volume of blood collected from each infant at each time point was 8ml. PBMC were isolated and cryopreserved in liquid nitrogen in 1-3 separate vials. Based on pilot studies conducted with non-case samples from the TB20 study the median expected recovery of viable cells from 1-2 thawed vials is 17 million (range 2-32 million).

Supplementary Table 7: Assay identification for C-020-485 case-control correlates analysis: Intermediate selection of key assays/variables

	**Assay**	**Antigen/Cell population/cytokine**	**Notes**
1	MGIA	BCG	Log growth in sample tube/log growth in control tube (possible measure of vaccine response)
2	ELISPOT	85A peptides	SFC/million PBMC (measure of vaccine response)
3	ELISPOT	BCG	SFC/million PBMC (possible measure of vaccine response)
4	ELISPOT	PPD	SFC/million PBMC (possible measure of vaccine response)
5	ELISPOT	10.3/10.4	SFC/million PBMC
6	ELISPOT	CMV	SFC/million PBMC
7	ELISPOT	EBV	SFC/million PBMC
8	ELISPOT	Flu	SFC/million PBMC
9	Flow Cytometry	T cells	% of Live CD3+ lymphocytes
10	Flow Cytometry	CD4+ T cells	% of Live CD3+CD4+CD8- lymphocytes
11	Flow Cytometry	CD8+ T cells	% of Live CD3+CD8+CD4- lymphocytes
12	Flow Cytometry	B cells	% of Live CD3-CD19+ lymphocytes
13	Flow Cytometry	Monocytes	% of Live CD3-HLA-DR+CD14+ CD16-
14	Flow Cytometry	Inflammatory monocytes	% of Live CD3-HLA-DR+CD14+ CD16+
15	Flow Cytometry	Regulatory T cells	% of Live CD3+CD4+CD8- CD25+CD127- lymphocytes
16	Flow Cytometry	Monocytes/lymphocytes	% of Live CD3-HLA-DR+CD14+ CD16-/ % of Live CD3+CD4+CD8- lymphocytes
17	Flow Cytometry	Inflammatory monocytes/lymphocytes	% of Live CD3-HLA-DR+CD14+ CD16+/ % of Live CD3+CD4+CD8- lymphocytes
18	Flow Cytometry	other -possible gamma delta T cells	% of Live CD3+CD4-CD8- lymphocytes
19	Flow Cytometry	Other – possible natural killer cells 1	% of Live CD3-CD19-CD14- lymphocytes
20		Other – possible natural killer cells 2	% of Live CD3-CD19-CD14-CD16+lymphocytes
21	Flow Cytometry	Activated CD8+ T cells	% of Live CD3+CD8+HLA-DR+CD4- lymphocytes
22	Flow Cytometry	Activated CD4+ T cells	% of Live CD3+CD8-HLA-DR+CD4+ lymphocytes

Supplementary Table 8: Description of outliers

Day	SUBJID	Variable	Value	assay type	% live cells	Investigation	Retain yes/no		
0	50242	E.BCG	-0.69315	elispot		viability low (61% and PH	no	6 elispot excluded, 5 retained	
0	50543	E.BCG	-0.69314	elispot		viability low (51%) and PH	no	6 flow retained, non-excluded	
0	50543	log(E.BCG)	-0.69314	elispot	50.8	PHA<1000	no	ed and 1 excluded	
0	50242	log(E.BCG)	-0.69315	elispot	57.4	PHA<500	no	19 outliers	12 retained
0	50599	log(E.BCG)	-0.69314	elispot	63.7	HIGH BACKGROUND EXCL	no		7 excluded
0	50599	log(E.PPD)	-0.69314	elispot	63.7	HIGH BACKGROUND EXCL	no		
0	50842	E.PPD	-0.69314	elispot		viability low (62%) but PH	yes		
0	50842	log(E.PPD)	-0.69314	elispot	62.8	ok low viability	yes		
0	51069	X85A	23.33333	elispot	74.6	ok	yes		
0	53439	X85A	48.33333	elispot	78.6	ok	yes		
0	52569	X85A	55	elispot	84.4	DAY 28 and DAY 7 WRON	yes		
0	53353	C.MON	-1.427097	flow		ok requested Iman to loo	yes		
0	53353	CML	-5.649561	flow		ok requested Iman to loo	yes		
0	52891	I.MON	-4.017384	flow		ok requested Iman to loo	yes		
0	53353	I.MON	-4.017384	flow		ok requested Iman to loo	yes		
0	53353	IML	-8.239827	flow		ok requested Iman to loo	yes		
0	52891	log(IML)	-8.228030	flow		ok requested Iman to loo	yes		
0	51971	M.BCG	-1.80672	MGIA		replicates ok, viabilty ok.	no		
0	51898	log(M.BCG)	-1.80672	MGIA	80.9	batch 10 incorrectly ente	yes		
28	50242	log(E.BCG)	-0.69315	elispot	53.6	PHA<500	no		
28	50344	log(E.BCG)	-0.69314	elispot	61.4	HIGH BACKGROUND EXCL	no		
28	50842	log(E.BCG)	-0.69314	elispot	65.7	batch 10 incorrectly ente	yes		
28	51093	log(E.BCG)	-0.69314	elispot	76.2	ok	yes		
28	52687	X85A	140	elispot	52.3	ok low viability	yes		
28	51295	X85A	81.66667	elispot	76.3	ok	yes		
28	53735	X85A	96.66667	elispot	78.3	ok	yes		
28	52462	X85A	110	elispot	79.9	ok	yes		
28	51898	X85A	83.33335	elispot	80	ok	yes		
28	51261	X85A	150	elispot	81.1	ok	yes		
28	50263	X85A	106.6667	elispot	81.7	ok	yes		
28	51024	X85A	83.33333	elispot	83.4	ok	yes		
28	50830	X85A	113.3333	elispot	84.9	ok	yes		
28	52044	X85A	356.6667	elispot	85.7	ok	yes		
28	50833	NK.16NEG	37.8	flow	64.2	Iman checking machine	no		
28	50110	A.CD4	43	flow	53.8	ok low viability	yes		
28	51841	A.CD4	33.90001	flow	61.2	ok low viability	yes		
28	51807	C.MON	29.9	flow	80	ok	yes		
28	51807	CML	0.892537	flow	80	ok	yes		
28	50110	GAM.DEL	7.72	flow	53.8	ok low viability	yes		
28	50141	GAM.DEL	9.77	flow	73.4	ok	yes		
28	51841	I.MON	10.20002	flow	61.2	ok low viability	yes		
28	53169	I.MON	7.48	flow	65.2	batch 10 incorrectly ente	yes		
28	51807	I.MON	7.12	flow	80	ok	yes		
28	52998	I.MON	10.5	flow	80.6	ok	yes		
28	51893	I.MON	7.01	flow	84.2	ok	yes		
28	51841	IML	0.211618	flow	61.2	ok low viability	yes		
28	53169	IML	0.187	flow	65.2	batch 10 incorrectly ente	yes		
28	51807	IML	0.212537	flow	80	ok	yes		
28	52998	IML	0.221519	flow	80.6	ok	yes		
28	51893	IML	0.135067	flow	84.2	ok	yes		
28	52687	NK.16NEG	17.2	flow	52.3	ok low viability	yes		
28	50110	NK.16NEG	12.3	flow	53.8	ok low viability	yes		
28	50155	NK.16NEG	15.10001	flow	61.1	ok low viability	yes		
28	50258	NK.16NEG	12.4	flow	64.3	ok low viability	yes		
28	50153	NK.16NEG	12.2	flow	65.3	batch 10 incorrectly ente	yes		
28	50190	NK.16NEG	15.1	flow	67.8	ok low viability	yes		
28	53314	NK.16NEG	16.8	flow	67.8	ok low viability	yes		
28	30044	NK.16NEG	12.9	flow	71.6	batch 10 incorrectly ente	yes		
28	50251	NK.16NEG	14.5	flow	73.9	batch 10 incorrectly ente	yes		
28	52456	NK.16NEG	20.4	flow	76.4	batch 10 incorrectly ente	yes		
28	51971	NK.16NEG	17.1	flow	76.5	batch 10 incorrectly ente	yes		
28	53747	NK.16NEG	13	flow	77.2	incorrect ID number now	yes		
28	51778	NK.16NEG	13.8	flow	77.5	batch 10 incorrect NOW	yes		
28	52190	NK.16NEG	23.7	flow	78.1	batch 10 incorrectly ente	yes		
28	51790	NK.16NEG	18.6	flow	78.5	batch 10 incorrectly ente	yes		
28	51832	NK.16NEG	25.5	flow	79	batch 10 incorrectly ente	yes		
28	51898	NK.16NEG	24.9	flow	80	batch 10 incorrectly ente	yes		
28	52743	NK.16NEG	24.7	flow	80	batch 10 incorrectly ente	yes		
28	53153	NK.16NEG	14.1	flow	80.7	batch 10 incorrectly ente	yes		
28	53202	NK.16NEG	15.7	flow	81.5	batch 10 incorrectly ente	yes		
28	53430	NK.16NEG	16	flow	82	batch 10 incorrectly ente	yes		
28	51116	NK.16NEG	21.9	flow	82.7	batch 10 incorrectly ente	yes		
28	53710	NK.16NEG	16.4	flow	84.4	batch 10 incorrectly ente	yes		
28	50009	NK.16POS	21.3	flow	77.8	ok	yes		
28	50208	T.LIVE	50	flow	50	ok low viability	yes		
28	52687	T.LIVE	52.3	flow	52.3	ok low viability	yes		
28	50349	T.LIVE	52.9	flow	52.9	ok low viability	yes		
28	50242	T.LIVE	53.6	flow	53.6	ok low viability	yes		
28	50110	T.LIVE	53.8	flow	53.8	ok low viability	yes		
